# Supplementary material for: Ploidy level enhances the photosynthetic capacity of a tetraploid variety of Acer buergerianum Miq
Source: PeerJ. 2021 Dec 16;9:e12620. doi: 10.7717/peerj.12620 (PMC8684723; doi:10.7717/peerj.12620)
Supplement: Supplemental Information 1 — Putative gene ID and sequence information. [file peerj-09-12620-s001.docx]

Table S1. The sequences of 9 candidate genes

| Putative gene ID | Genes sequence |
| --- | --- |
| CL6641.Contig2 | ATGGAGAATCCGCAACAGGAGGTCGTGAGAGTTCGGTGCCAGCGAATTGGCTGCGACGCCATGTTCACCGAAGACGACAATCCCGAAGGTTCCTGCACTTTCCACGATTCGGGACCTATATTTCATGATGGGATGAAAGAGTGGAGTTGTTGCAAGAAAAGAAGTCATGATTTCAGCTTATTTTTAGAAATTCCAGGATGTAAGACAGGTAAACACACAACTGAAAAACCAGTGTTAAAAAAGGTGGTTGCTGCTCCAAAAGCTTCATTACCTGCTGCTTCACAAGCTCCTGCGTCCAGTACACCATCAAAGGAAATTTGCTCTAGATGTCGACAGGGTTTCTTTTGCTCAGATCATGGTTCACAAGCCAAGGGTCTGAACCAGTCAGCTGCAGTTGCGCCTGCTGAAAATTATTTCGAAGTTCAGGCATCTCCTGCTCCAGTTAAGAAGATAGTTGATATAAACGAGCCACAAATCTGCAGAAATAAGGGATGTGGTAAGACATTCAAAGAAAAGGATAATCATGAGACGGCATGTATGCACCATCCTGGGCCTGCTGTTTTTCATGATCGGATGAGAGGGTGGAAGTGCTGCGACATTCATGTAAAAGAATTCGATGAGTTCATGAGCATTCCACCGTGCACCAAGGGATGGCACGACGCAAATCCAGGATCATGA |
| CL7423.Contig2 | ATGGGCATTACCTCCAACTTGGCCTCCTTCTTTTCTTCTTTTTTTTCTTCTTTCTTTTCTTCGACGCTCACCAAATCGGCATACCCTAGTTTCTTCCTCAACAGCAGCGTTAGCACCGCCGGGTCGAACCCCTCTCCGGTCACCTCGATGTTATCCCCTTTCCACGCCGCGGATTCCACACCAGAAGCTCCAACTACAATCTTCAAGGCCTTAGGGCGGGACTTTTGGCCGTTCATTGTAACTTTGATCACCACCTTTTGCTTCATCGTCTGGTTTTTTGTGATTGTTGAAGATGATACTCTGAACACAAATTGGCTTGAATCAGATTGGAACTGTATGGAGTACTATTGTTGTGGTTGTCAAAAATGGACTTGTGTGGTTTTTAATTTG |
| Unigene5873 | CAAAACTCAGAGATAGAGAGAGAGAGAGGGTGTAGTCACCGGACAGTAGAGAACCGCAGTGAAGAAATTAAAAAAAAAATAAAAAAATCATATTTTTACATGGGTGGTGGTGGTGGTGGTGGTGCTCTGTTTTCTGAAAACATGGTTGCAGTGAACAGGAAAGACAAAGTGATCGAAGAGCTCGTTCAAGGGAAAGAATTCGCGGGTCAGCTTCAGGTTCTGCTTCTCCAGCAACCCTTTGGAGGAGATGGGTCGTCGGCGGCGGCGGAGGAGCTGACGGGGAAGATCTTGAGGTCTTTT  ACAGAGACTCTATCTGTGCTGGGTTGTGGTGGTGAGACTACGTCGTCTGCTGGGGAGGTGGTGGAGGAGGAAGGTGATTCGCTGGTTTCCGGTGGCCGGAGATCGGAGGAATCCGGCGAGAGTAGGAAGAGGTCTATGGGTCCCAAGGGGAGGAGAGGTTGCTATAAGAGAAAGAAGAATTCAGAGACATGGAGAATGATGACACCTATCATTGAAGATGGTTATGCATGGAGAAAGTATGGGCAAAAAGAAATTCTCAATACTAAATATCCAAGGAGCTACTTTAGGTGTACACACAAGTATGAACAAGGTTGCAAAGCTACAAAACAAGTTCAAAAAATGGAAGACAACCCACAAATGTATGAGACCATATACATTGGCAAACACACTTGTAGGAGCCAAATGTCACTCACATCAAAACCACAAATGATCATCACAGACATTAATGGTGGACCTTGGAGGAGTTCTTCTTCTGTGATCAGCTCTCCATCATCATCAACAGCAGTGGTAGTAGTTAAACAAGAATACAATCTCAAAGAAGAAATCAGTAATGGTGGTGGTGGTGATGATTTTTCAGAAAACCTATCATCATCTCATCATCTTGGTGACCATGAGCAGCAGATGGAATATTTTTCCATGTGGGATGATCTGGACCCTTTAAATCATCATCATCAAGTTTCTATCACCACCACAACTTGCCATCAGGAAAATTTCCACATTGATTTTGATAGTGAACTTTATTTTGATGAGAGTGATTTATTCCAGCTAGCTAGCTAA |
| CL1401.Contig2 | ATGGCACAGCTCGCAATTAAAGCAGCAATAAGTATACTTGGTAGTATAGTCGAACGTGTAGATCTAAGAAGCTCACAGTTTGATGTCGATGCAGCGAAAAATTTCATGAACACCATGAAAGCTTACCTGATTGATACAGAAGGAAGAGAAGGCACCGAAGGCTTCAAGGATCGCGTTCAACAAGTGCGTGATGTGGCCTACGAGATCGAGGATGTTATTGAAGAGTTCATGCTTGAAGTGCCTGAACACTTTCATGAGCATAGAATCACCAAATATCTTCATGATTCTGGTCATTTTGTCAAAGACCAAATGGCTGTCCAAAGATTGTCCTCGCGTATGGAGGCTATCGAGGTCAAGATTCATAAAATAAAAGACATGGATTCATTTCGCATTTTCCCCCCTGAAGGAGCCTCGAGTTCTCGTGGTGGGGAACAAGAAAATGAATTAGTTGGCATTGAGAAATGCTACAACAATCTTTCCCCAAATCTCAGGTACTGCTTCTTGTACTTCTGTAACTTCCCTGCGAACTATTTTGTTACGCGTGGAAGACTTTTTCGCTTGTGGATAGCTGAACGGTTCATAGAGGAGGAGGGAAGGAACAAGACAAAGGAGGATGTGGCTGATGAATACCTCAACGAACTCATTGAAAAGAACTTGGTTTATGTCTATGGTAGACGATTTGGGGTTCCTCAACCTGTGCGAGACTTCATCCTTGACAAGTCTGAAATGAAAATTTTCTGCACCGTTCTGCCGAGACCAAACGGTACAATTCCAATTGAGAAAAGCAGGCGCTTATCTCTTCACAATGGATTCACCGATTCCTTACGAAGCAAGGACTTATCTTGTGTTCGTACCCTGATGACATTCAGGAGAGACTCCGAATTAAAAGCTGAGGAATTGCTCAACAAATTTAGATTGTTGAGAGTTTTGGACTTTGAAGATTCAGGTTTGCAAACTTTTCCTGAAGAGGTGGTCAAACTCACACTCCTAAGGTATTTAAGTTTCAGGAATACAAACATAAAAAAAGTTCCTAGATGTATTAAGAAACTTCACAGCCTTGAGGCTTTGGACCTTAGACAAACATGTGTCAAGAAGTTGCCCAAGAAAATCCTGAAACTTCAGAATTTGTTGTATCTTTTAATGGACCAAAGACTAGGTGTTGATGAAGCTCGTGAAGGAGTCGAATTGCCTGCAGGATTTGAACGTTTAAAATCATTACGGAAGCTGTCTCTTGTCAAGGCAAACAAAGAGAATCGGAGGATCATAAGAGAGTTAGGAAATTTGATTCAACTCAGGAAACTAGGGATCACAGAGCTTGAAACGAAAGATGGAATGGATTTCTGCGCGTCCATCCAAAGCATGGAGTATCTTTCCTCTTTATCGGTGACAGCAGCCTACAGAGATGATGAAGAGGGGCTTCTTGATCTGGATCATGTAAGGAAACCTCCTCCTTTCCTTCACCGTATAAGTTTAGGAGGTCGTCTACAAGACATTCCACAGTGGATTAGCTCATTGCAGAGTCTTGCAAGTATATGTCTTAAAGGTTCCAAATTAGAGAATAGCCCTCTTGATGCCCTCCAAGCTTTGCCTTGTCTTGTGGAGCTCAGGCTGGTTGATGCATACGTTGGGGAAGTGTTGGAATTTGAACCCGGGTGTTTCCTGGAACTAAGGGTGTTATATCTTCAACAATTGGATGGATTACAAAAGGTTTCAGTGAAGACCCGCAACGCACTTCCTAAACTTCAGAGGTTAGTTTTCAGAGGATTTCAAAGGTTGCCAAAGTTTCCTTCTCGATTCCAGCAACTAATTAAAGAGGAGTAA |
| CL2285.Contig2 | ATGGGAGGCAAAAATTATCTTGGTTTTGTGATATTTTTGAGTGTTTTAGGATTTATAGGCTCATGTCAAGCTCAGTTGCAGATGGGGTTTTACTCTAAGAGCTGCCCAAAAGCTGAGACGATTGTGCTTGATTATGTCAAAAAACACATCCCAAATGCACCATCACTCGCTGCAACTTTCATCAGAATGAATTTCCATGATTGTTTTGTCAGGGGTTGTGATGCTTCTGTGCTTCTAAACTCAACTTCAGGAAACGCTGAGAGGGATGCAGTTCCAAATCAAACACTGAGAGGTTTCGGCTTCATTGACGCTATCAAGAGCCTGCTTGAAAAAGAATGCCCAGGCGTAGTTTCATGTGCAGATATCATTTCTTTAGTTTCAAGAGACTCCATTGTACTCACTGGAGGTCCTTCCTGGCAAGTTCCAACAGGAAGAAGAGATGGGTCAATCTCCAGGAGCTCAGAAGCTACCAGCAACATCCCATCTCCATTTTCAAACTTCACAACTCTCCAGACACTTTTTGCTAATCAAGGACTTGATTTGAAAGACTTGGTTTTGCTCTCCGGTAAGTATCAATTTTTCTGGAAACAAAATCCAGAACTCGGTTCGAGCCCAGACCTCTTCCAAAATCTTGAAATATTGTAA |
| CL3396.Contig3 | ATGGTTTCCAAAGCACTCTTCTTCTTTGCTTTGTTGTCCTTCTCAGCTGTGTCTCTCAGGCCAGCGGCTTCTGCAGAAAATGAAGAAGACCCTGGTCTTATGATGAGCTTCTACAGAGATACATGCCCTCAAGCTGAGGACATTATCAAAGAACAAGTCAAGTTGCTTTACAAGCGCCACAAGAATACTGCATTCTCTTGGTTGAGAAACATCTTCCATGACTGTGCTGTTCAGTCATGTGATGCTTCATTGCTGTTGGACTCAACAAGGAGAAGCTTGTCTGAGAAGGAAACAGACAGGAGCTTTGGGATGAGAAACTTCAGGTACATTGAGACCATCAAAGAAGCTCTTGAGAGGGAGTGTCCTGGAGTTGTCTCATGTGCTGATATCCTTGTGTTGTCTGCTAGAGATGGCATTGTTGCTCTAGGAGGTCCTTACATTCCTCTGAAGACAGGAAGAAGAGATGGCAGGAAGAGCAGAGCAGATATCCTTGAAGAATACCTCCCTGATCACAATGAGAGCATGTCTGTTGTCCTTGACAGGTTTTCAGCCATCGGTATTGACACCCCTGGAGTTGTTGCTCTGCTAGGAGCTCACAGTGTTGGAAGAACCCACTGTGTGAAGTTGGTGCACAGATTGTACCCAGAGGTTGATCCAGCTTTCAACCCTGACCATATTGACCACATGCTCCACAAGTGTCCTGACCCAATCCCAGATCCCAAGTCTGTGCAGTATGTGAGAAATGACCGTGGCACACCCATGATTCTAGACAACAACTACTACAGGAACATTTTGGACAACAAGGGTTTGATGTTGGTGGATCACCAACTAGCTGTAGACAAGAGGACCAAACCATTTGTGAAGAAGATGGCCAAGAGCCAAGCTTACTTCTTCAAGGAGTTTTCAAGAGCCATTACCATACTCTCTGAGAACAACCCTCTCACTGGTACAAAGGGTGAGATCAGGAAGCAGTGCAATCTTTCCAACAAGCTGCACTAG |
| CL6251.Contig1 | ATGAAGCTTGAAGAATATTACACTGCTAAATCAGCCCTTGAAAAAGGTAGCTCTTTGGCAGAGAATGACTCGAGATTTACCAAGTTGATTGAAGAATGTGATAAGTCTATTGCTGAGGAGAACAACGATCTGGCTAAGCCATTGTCACCCGATTTGACAACAAGCTCCACTCCTTTAACGAAGCCAGTCACATCCATCAAACCAAAATACAGACATGAATTCTACCAAAAGGCGGATGAAGTGGTTGTGACCATTTTTGCAAAAAGAATACCAGCAACGAGTGTGACTATTGAATTTGGCGAGCAGATCTTGAGTGTTACCATTGATGTCCCTGGTGAAGATGCTTATCATTTTCAACCTCGATTGTTTGGAAAGATAATACCTGGGAAGTGCAGATATGAAGTATTATCAACCAAAGTTGAAATCCGTCTTGCGAAAGCTGAAGCTATTAACTGGACATCTCTTGAATATAGCTCGGAAATTTCAGTTTTGCAGAAAGTAAATGTGCCACCAGTTGCATCTCAGAGGCCTTCATATCCATCTTCGAAGAAAACAAGAGACTGGGACAAGCTGGAAGCCGAAGTAAAGAAGGAGGAAAAAGAAGAGAGACTAGATGGTGATGCTGGTGTGAACAAGCTGTTCCGGGACATTTATCAAAATGCAGATGAGGACATGAGAAGAGCTATGATGAAATCTTTTGTGGAATCAAATGGAACGGTGCTTTCAACGGACTGGAAAGATGTGGGTGCAAAAAAGGTTGAAGGTAGTGCTCCCGAGGGTATGGAGGTGAAGAAATGGGAGTACTAA |
| CL335.Contig5 | GACTTTACCCAATCTTTTCCTCTGCAAAATTCACAGACAGAGAGAATGAGTGGAGAAGGGAAAGTGGTGTGTGTAACAGGAGCTTCGGGTTACATAGCTTCGTGGCTGGTCAAGCAATTACTCCAACTTGGTTACACTGTCAAAGCCACTGTTCGTGACCCAAATGATCCCAAGAAGACAGAACATTTACTTGCACTCGATGGGGCTAAAGAAAGACTTCATTTTTTCAAAGCAAGCTTGTTGGAAGAAGGATCTTTTGATTTTGCAGTTGATGGTTGTGATGGTGTTTTCCATACAGCCTCCCCTGTCCTTCTATCCTCCAAAGTCTCTCAGGCAGATGTAGTTGACCCTGCATTGAAAGGAACACTTAACGTTCTTAGATCATGTGCTAAAGTTCCATCTATCAGAAGGGTCATCTTAACATCTTCTACTGCAGCAGTTTTATTCAACGGAAAGCCTCTTACTCCTGATGTTACGGTTGATGAAACTTGGTTTTCTGATCCGGCTTTTTGTGAGAAATCAAAGCTTTGGTATATTCTTGGGAAAACCTTAGCTGAGGAGGCTGCTTGGAAGTTTTCAGAAGAGAACCGAATCAATATGGTTGCTATAAATCCAGGCATGGTAATCGGTCCTCTCTTACAGCCAACACTTAACGACAGTGTCAAGCCTATTCTGAATCTCATAAAAGGAGTACCATGTTGGACTGATGGATGGGTTGATGTTAGAGATGTGGCGAAGGCACATATTCAAGCATTTGAGAATCCCACAGCATGTGGTAGATACTGTCTGGTTGAACGAGTTGTGAACCATTTTGAAATTTTTAAGATTTTACGTGGGCTTTATCCTGATTTAAATCCTCCCGAAGAATACTCGGAAGAGAAAGCTTCCGAACCAACCTACCGGGTGTCCAAAGAGAGAGTGCAAAGTCTAGGCATCACCTATATTCCCTTTGAAGTGAGTTTGAAGGATACCGTTGAAAGCTTGAAGGAGAAAAACTTAATCAATGCCTGA |
| CL5835.Contig1 | CTCCCTTTCACCAGCTCCCCTCTACTACCATCACCTACTTTTATATTACCTCAACCAATACTAAAACCAAATCAAAAAATTAAATTTAAAAAGAAAAAAAAAAAAAAAACCGAACTCTCACCTTCTCTTTTGTTTCCCAAAATCTTCTCTCACTATCTTTCTGTGTTGACAACAACAATGGAGGGCAAAGAAGATGAACATGAATTCATATTCCGATCCAAACTTGCTGATATTTACATCCCAAATCACCTCCCTCTACACACATATTGCTTTGAAAACATTTCTCAATTTAAAGATCGTCCCTGCATCATCAACGGCCCCACCGGTGACATCTACACATACGCTGATGTTGAACTCACTTCACGCAAAGTTGCCGCCGGTCTCCACAAATCCGGCATCAAAAAAGGTGACGTTGTCATGCTTTTGCTCCAAAACTGCCCTCAATTCGTGTTTGCATTTCTGGGTGCTTCCTATATCGGCGCCATAAGTACTACGGCGAATCCTTTCTACACTCCGGCAGAGATTGCAAAGCAGGCAACGGCTTCCAAGGCAAAGCTGATCATAACGCAAGCCCTATACGCCGAGAAAGTGAAGGACTTGGCCGAAGAAAGTGGATTCAAAATCATGACCATTGATTCATCTCCTGATCAAAATTGTTTACATTTCTCTGAGTTGACTAAGGACGATGAAAACGACATCCCCGCCGTGAAGATCAACCCGGACGACGTGGTTGCACTTCCTTACTCGTCGGGGACCACAGGGTTGCCTAAGGGAGTGATGCTGTCACACAAAGGCCTTGTCACGAGCGTGGCACAACAAGTTGATGGAGACAACCCTAATTTGTATTTTCACAAAGAAGATGTCATTTTATGTGTGTTGCCTTTGTTTCATATTTATTCGTTAAATTCGGTGTTGCTATGCGCTTTGAGAGTAGGCGCGGCCATCTTGATTATGCAAAAGTTTGAGATCGTTACATTGATGGAGCTTGTCGAAAAACATAAGGTCACAATTGCTCCCTTCGTCCCTCCAATCGTTTTGGCCATAGCCAAGAGCCCGGACCTCGATAAGTACAACCTTTCGTCCATTCGAACCGTCATGTCTGGCGCTGCGCCCATGGGGAAGGAGCTTGAGGATGCCGTGAGAGCTAAGCTTCCTAATGCCAAACTTGGACAGGGGTATGGAATGACAGAAGCAGGGCCAGTGTTGTCAATGTGTTTGGCATTTGCAAAGGAACCGTTTGAGATAAAATCAGGTGCGTGCGGGACCGTGGTAAGAAATGCAGAGATGAAGATTGTTGATCCTGATTCCGGTGCCTCCCTTCCTCGAAATCAATCTGGTGAAATTTGCATCAGAGGAAGCCAGATCATGAAAGGGTACCTAAATGATGAGGAGGCAACAGAGAGAACGATAGACAAAGATGGATGGTTGCATACAGGTGATATAGGGTTCATTGACGATGATGATGAGCTCTTCATCGTTGATCGATTGAAAGAATTGATCAAATACAAAGGCTTCCAAGTGGCTCCCGCCGAGCTCGAAGCTATGTTGATTGCCCATCCTAACATCTCTGATGCTGCTGTTGTCCCCATGAAAGATGAGGCCGCAGGAGAAGTTCCTGTGGCATTCCTTGTCAGATCAAACGGTTCTAAAATCACTGAGGATGAAATCAAGCAATACATCTCCAAACAGGTTGTATTCTACAAGAGAATTAGTAGGGTTTTCTTCACAGACACAATTCCAAAAGCACCATCAGGCAAAATCTTGCGTAAAGACTTAAGAGCAAAGCTGGCCGCTGGTCTGCCCAATTAG |
