# Supplementary material for: Ploidy level enhances the photosynthetic capacity of a tetraploid variety of Acer buergerianum Miq
Source: PeerJ. 2021 Dec 16;9:e12620. doi: 10.7717/peerj.12620 (PMC8684723; doi:10.7717/peerj.12620)
Supplement: Supplemental Information 2 — Putative gene, size, function and primer sequence information. [file peerj-09-12620-s002.docx]

Table S2. Primers used for the expression pattern of qRT-PCR products

| Putative gene | Size (bp) | Function | Forward primer (5'-3') | Reverse primer (5'-3') |
| --- | --- | --- | --- | --- |
| CL6641.Contig2 | 1220 | - | GCCATGTTCACCGAAGACGA | TTCTTGCAACAACTCCACTCT |
| CL7423.Contig2 | 677 | speckle-type POZ protein | ACGCTCACCAAATCGGCATA | GGCGTGGAAAGGGGATAACA |
| Unigene5873 | 1198 | DNA binding transcription factor activity | TGCATGGAGAAAGTATGGGCA | ACATTTGTGGGTTGTCTTCCA |
| CL1401.Contig2 | 2178 | ADP binding | ACAGGTTTTCAGCCATCGGT | CTGGGATTGGGTCAGGACAC |
| CL2285.Contig2 | 1758 | Peroxidase activity | GACGCTATCAAGAGCCTGCT | TGGAACTTGCCAGGAAGGAC |
| CL3396.Contig3 | 1508 | Heme binding | ACAGGTTTTCAGCCATCGGT | CTGGGATTGGGTCAGGACAC |
| CL6251.Contig1 | 918 | - | GGCGAGCAGATCTTGAGTGT | TCAGCTTTCGCAAGACGGAT |
| CL335.Contig5 | 1329 | Catalytic activity | TAGCTTCGTGGCTGGTCAAG | TTAGCCCCATCGAGTGCAAG |
| CL5835.Contig1 | 2060 | Catalytic activity | CGCCGAGAAAGTGAAGGACT | CTCCCTTAGGCAACCCTGTG |
| TUB |  |  | CTCGCTAACCCGCCTAAACA | ATGTCAAGTCCAGCGTGTGT |
