# Supplementary figures and images for: Ploidy level enhances the photosynthetic capacity of a tetraploid variety of Acer buergerianum Miq
Source: PeerJ. 2021 Dec 16;9:e12620. doi: 10.7717/peerj.12620 (PMC8684723; doi:10.7717/peerj.12620)

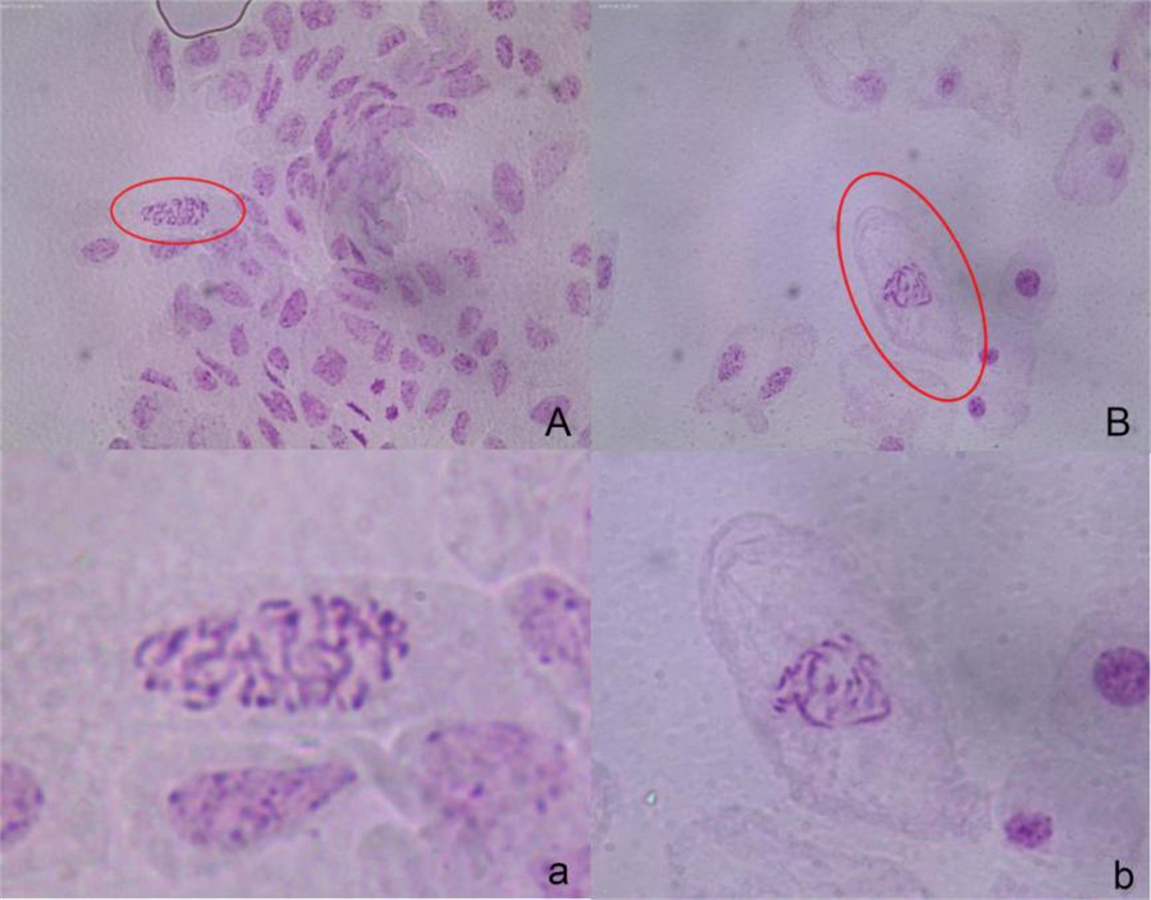

Supplement: Supplemental Information 7 — A and a: A. buergerianum X, chromosome number of diploids (2n = 2x = 26); B and b: A. buergerianum S, chromosome number of autotetraploids (2n = 4x = 52). Figures a and b are enlarged views of the red circled cells in Figures A and B. [file peerj-09-12620-s007.png]

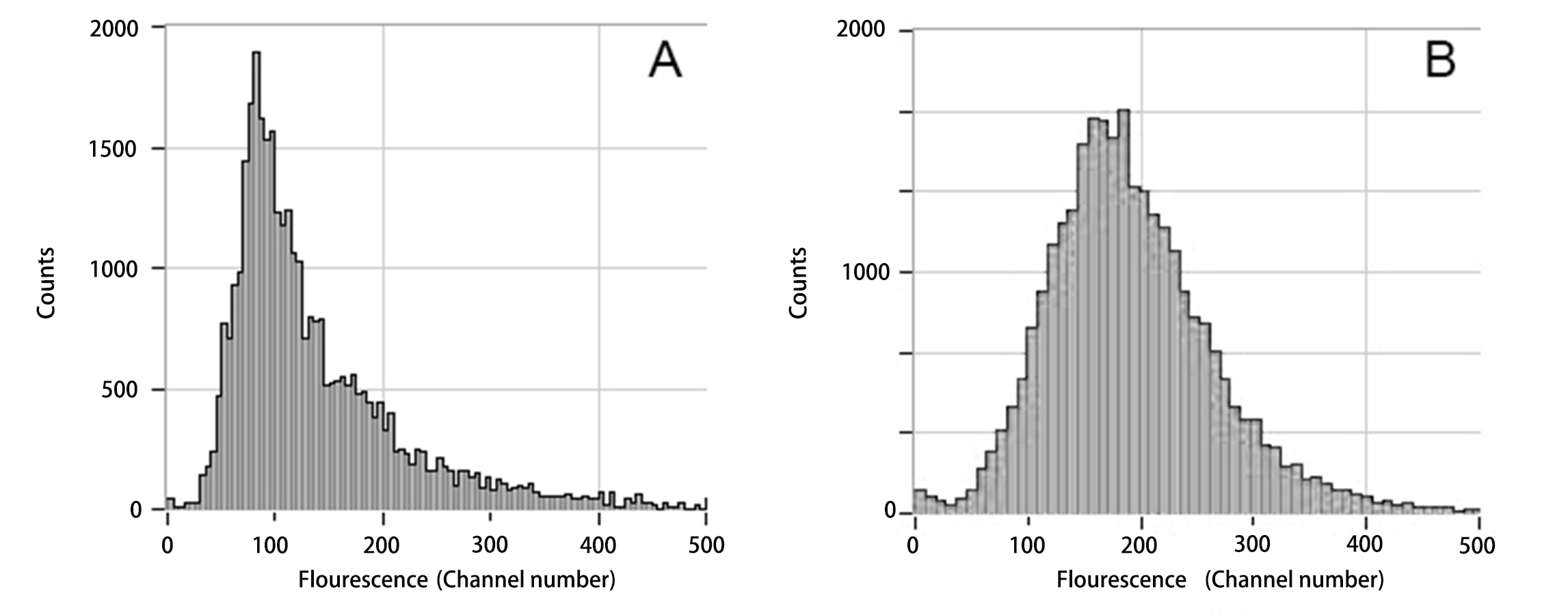

Supplement: Supplemental Information 8 — A: A. buergerianum S: DNA content of diploids (the main peak at channel 100); B: A. buergerianum X. DNA content of autotetraploids (the main peak at channel 200). The main peaks represent mature cells and the secondary peaks represent meristematic cells. [file peerj-09-12620-s008.png]

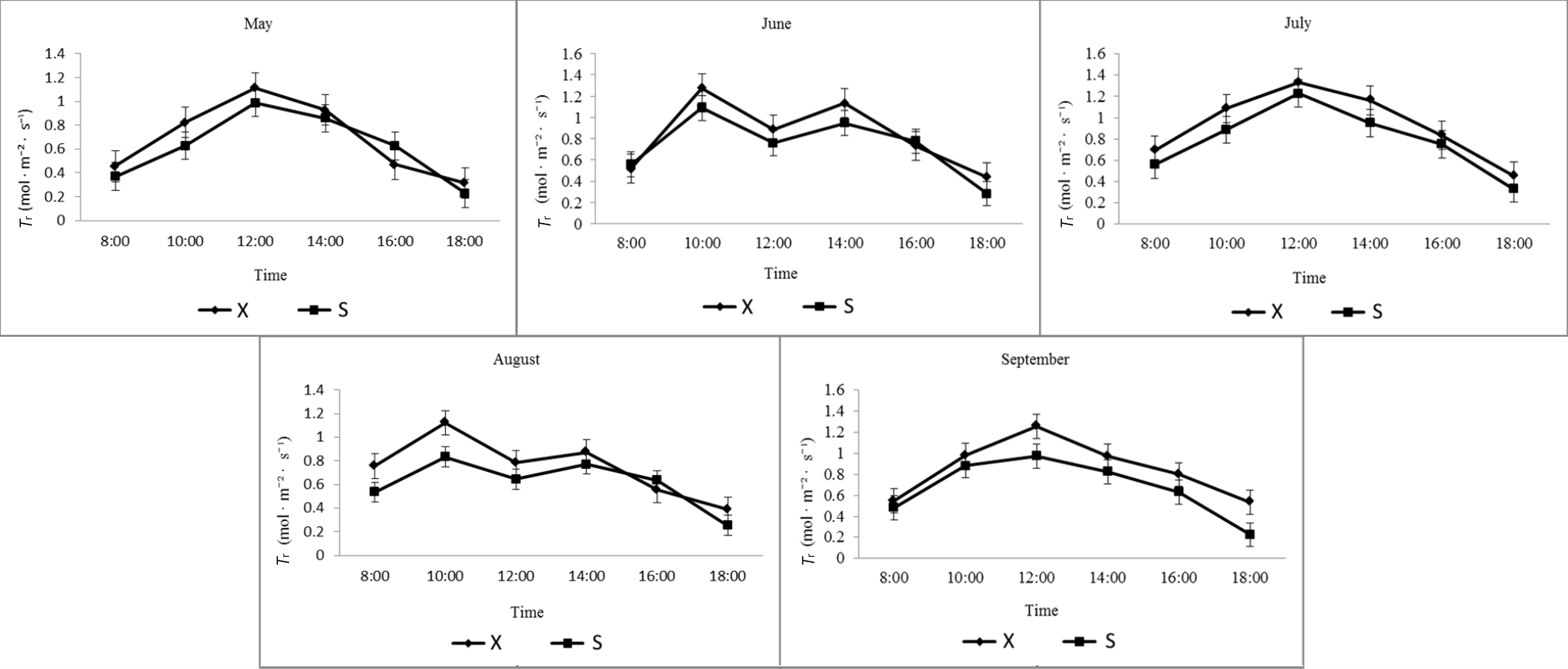

Supplement: Supplemental Information 9 — X: Tetraploid; S: Diploid [file peerj-09-12620-s009.png]

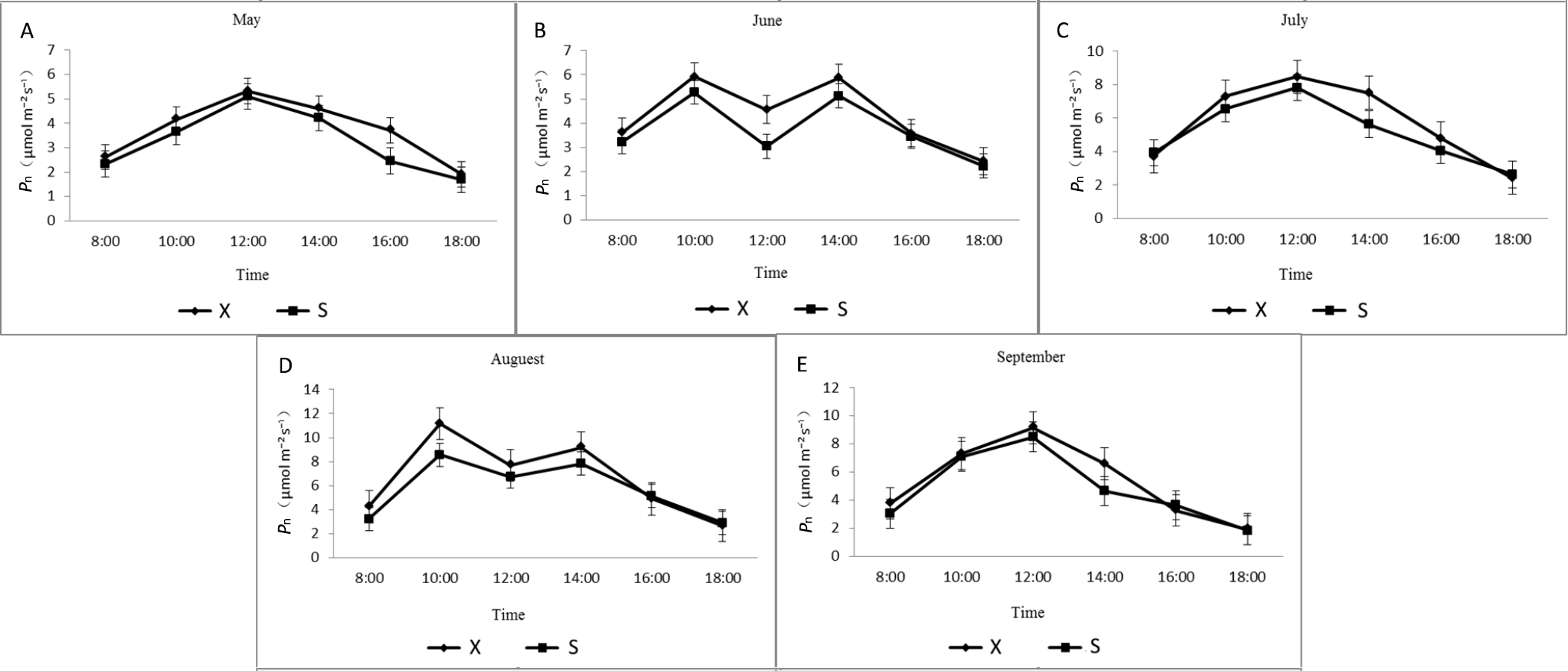

Supplement: Supplemental Information 10 — X: Tetraploid; S: Diploid [file peerj-09-12620-s010.png]

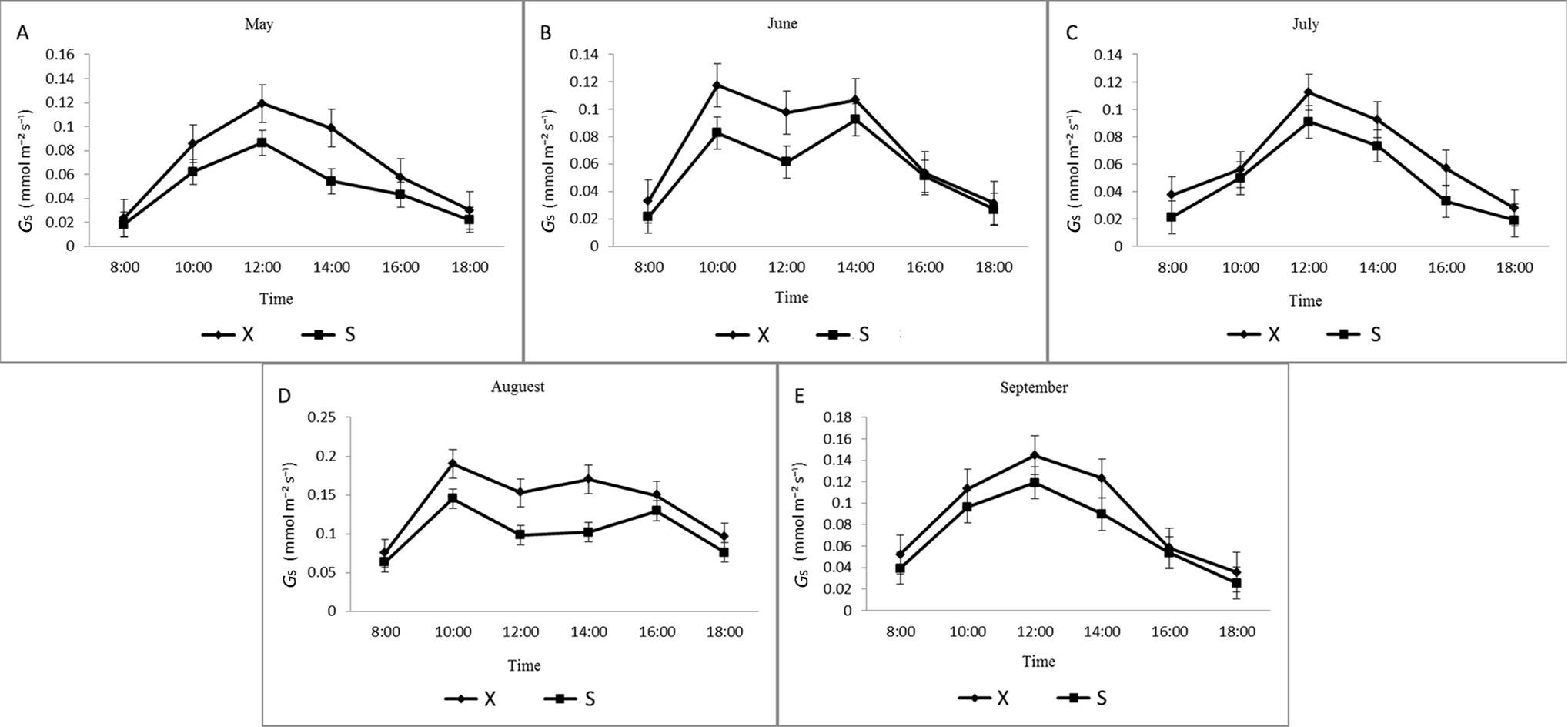

Supplement: Supplemental Information 11 — X: Tetraploid; S: Diploid [file peerj-09-12620-s011.png]

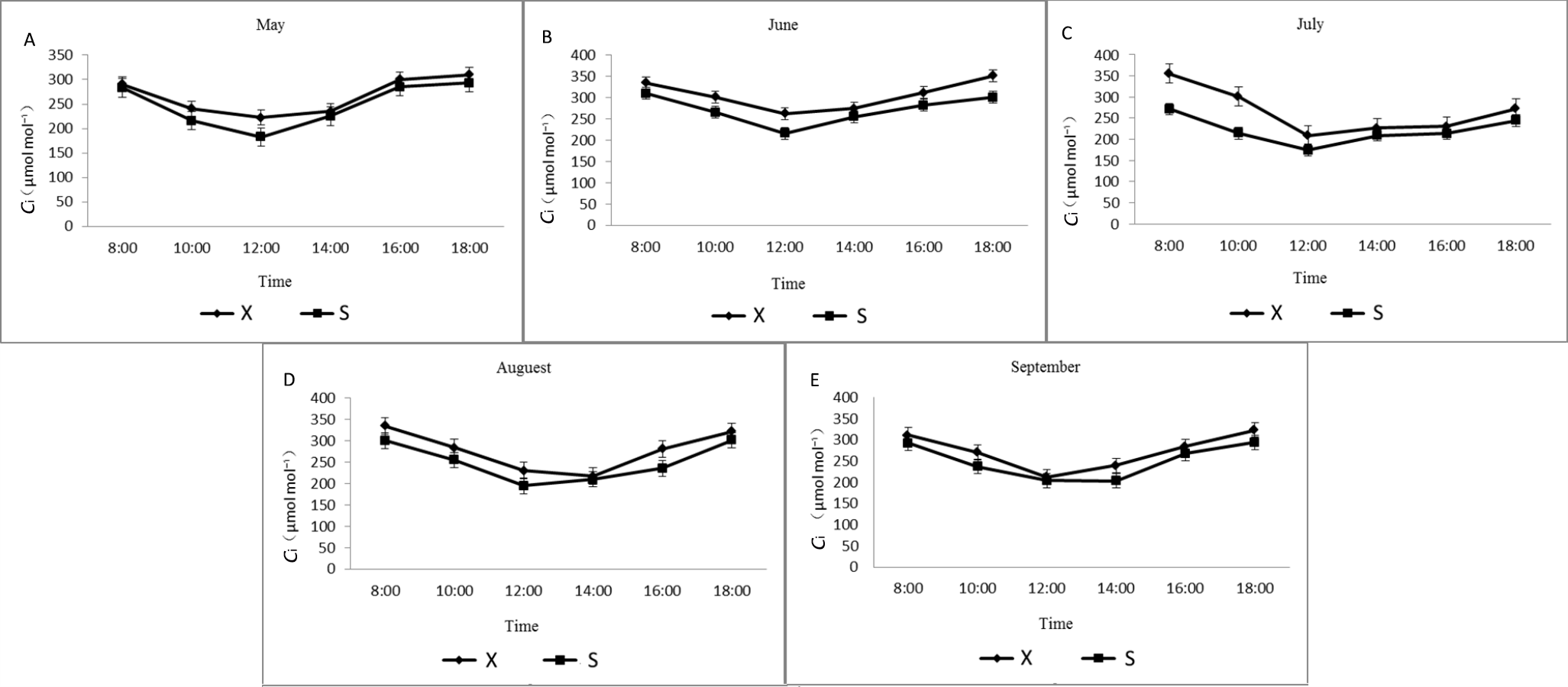

Supplement: Supplemental Information 12 — X: Tetraploid; S: Diploid [file peerj-09-12620-s012.png]

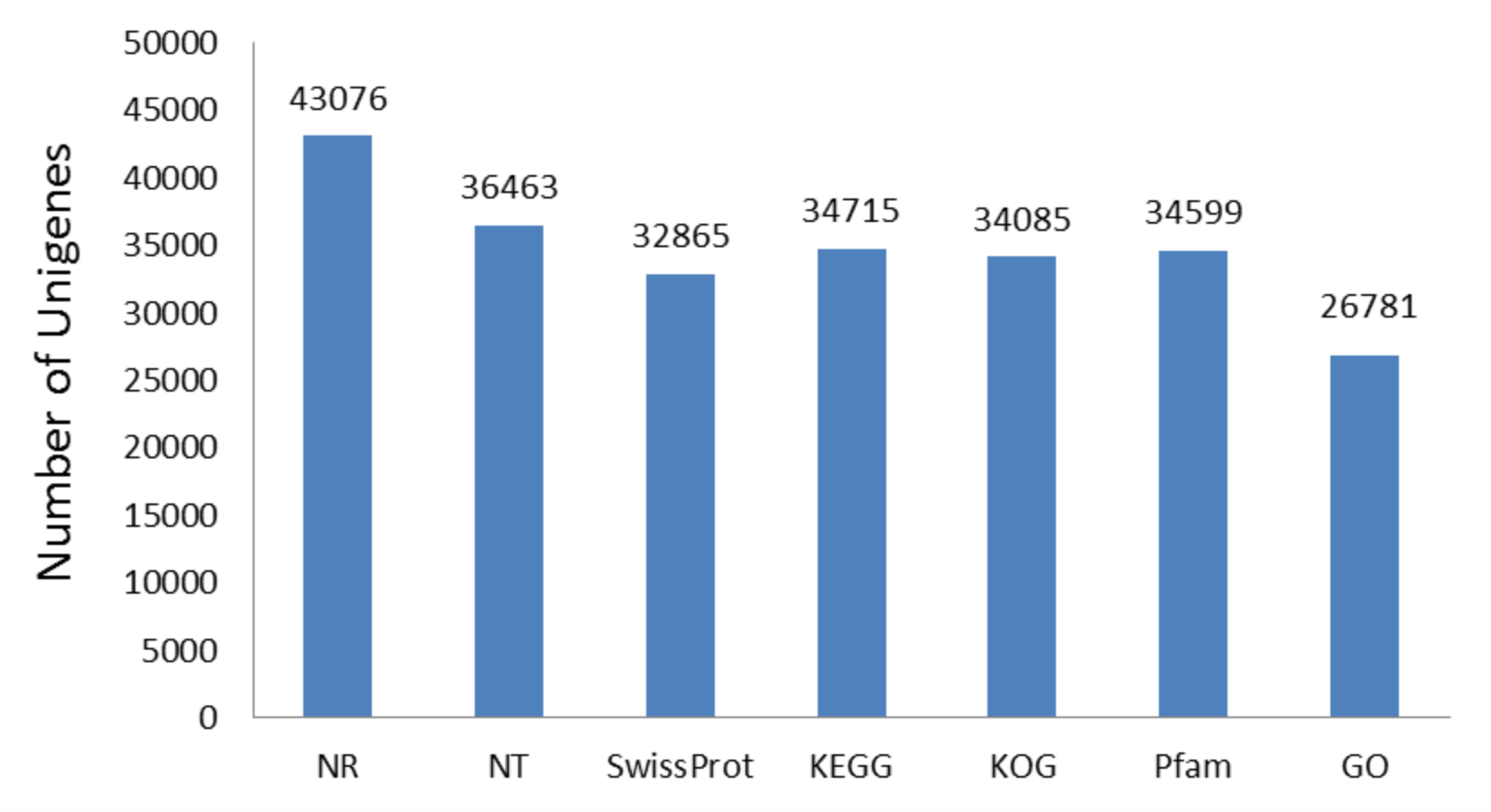

Supplement: Supplemental Information 13 — A total of 44665 Unigenes were annotated, accounting for 86.21% of all Unigenes. [file peerj-09-12620-s013.png]

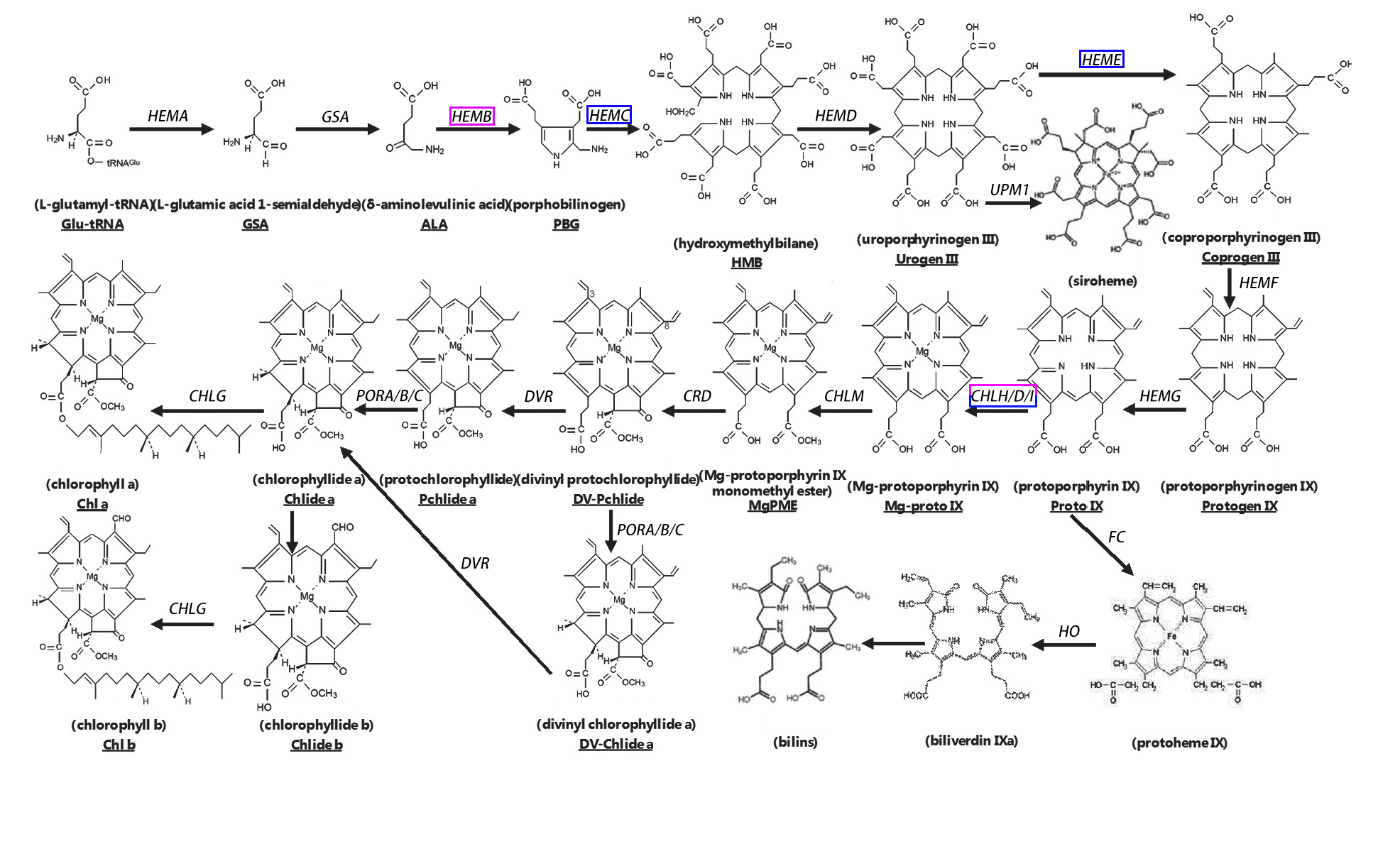

Supplement: Supplemental Information 14 — Up-regulated genes in A. buergerianum X are represented by purple boxes, and down-regulated genes are represented by blue boxes. [file peerj-09-12620-s014.png]

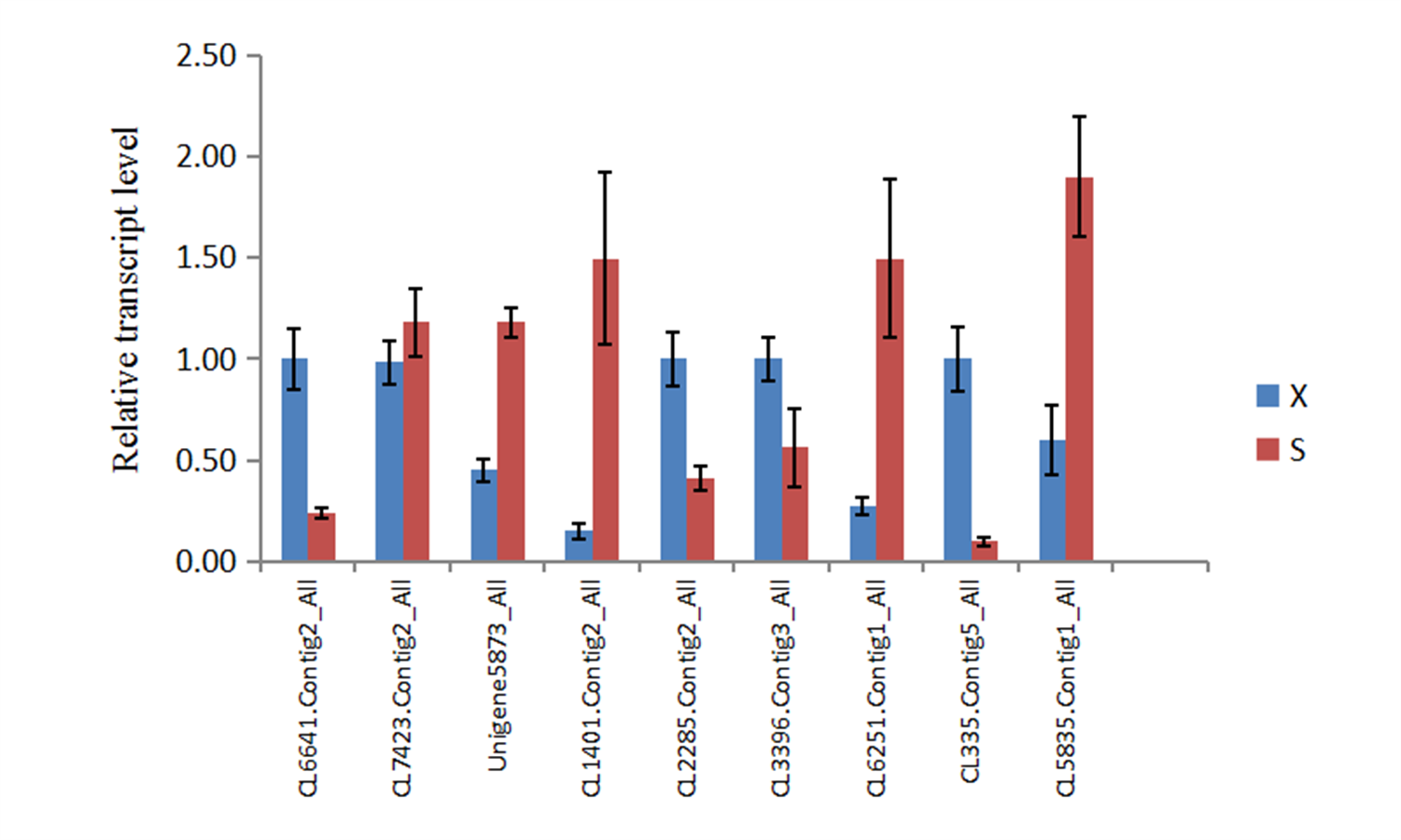

Supplement: Supplemental Information 15 [file peerj-09-12620-s015.png]

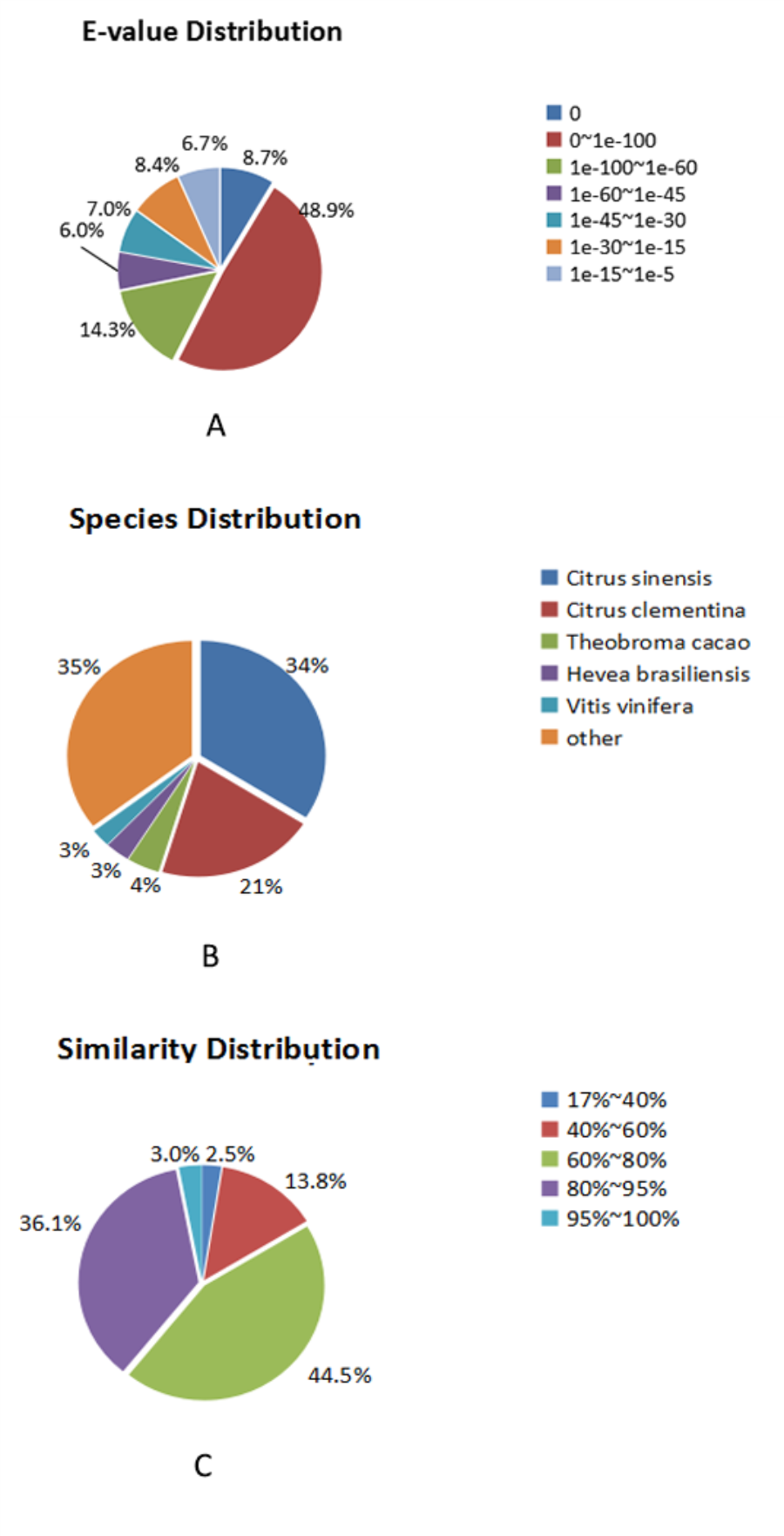

Supplement: Supplemental Information 16 [file peerj-09-12620-s016.png]

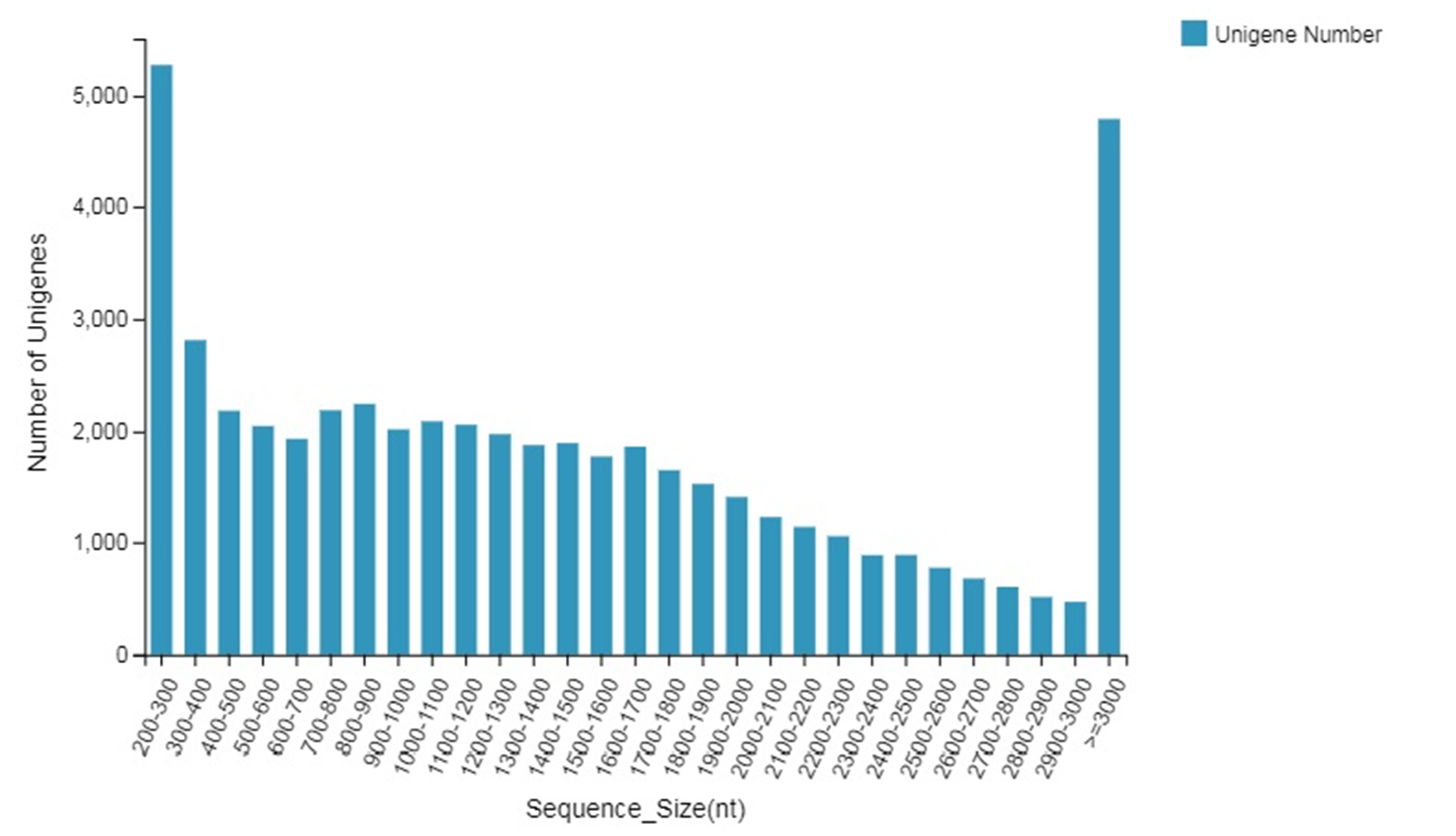

Supplement: Supplemental Information 18 [file peerj-09-12620-s018.png]
