# Supplementary material for: Ploidy level enhances the photosynthetic capacity of a tetraploid variety of Acer buergerianum Miq
Source: PeerJ. 2021 Dec 16;9:e12620. doi: 10.7717/peerj.12620 (PMC8684723; doi:10.7717/peerj.12620)
Supplement: Supplemental Information 21 [file peerj-09-12620-s021.docx]

Statistics on the results of sequencing X and S

|  | X1 | X2 | X3 | S1 | S2 | S3 |
| --- | --- | --- | --- | --- | --- | --- |
| Statistics of data production |  |  |  |  |  |  |
| Number of clean reads (Gb) | 7.00 | 7.12 | 6.73 | 6.91 | 6.63 | 6.90 |
| Total nucleotides (nt) | 7.00 | 7.12 | 6.73 | 6.91 | 6.63 | 6.90 |
| Q20 percentage (%) | 97.91 | 97.84 | 97.89 | 97.41 | 97.41 | 97.38 |
| GC percentage (%) | 41.69 | 41.37 | 41.67 | 41.46 | 41.36 | 41.52 |
|  |  |  |  |  |  |  |
| Unigene |  |  |  |  |  |  |
| Number of unigenes | 31592 | 35793 | 31998 | 34327 | 36701 | 33860 |
| Total nucleotides in unigenes (nt) | 40,858,989 | 46,287,414 | 41,267,382 | 44,779,162 | 46,183,329 | 43,952,421 |
| Average length of unigenes (nt) | 1293 | 1293 | 1289 | 1304 | 1258 | 1298 |
| Length of N50 (nt) | 1832 | 1841 | 1833 | 1850 | 1796 | 1843 |
|  |  |  |  |  |  |  |
| All-unigenes |  |  |  |  |  |  |
| Number of all-unigenes | 51807 |  |  |  |  |  |
| Total nucleotides (nt) in all-unigenes | 77,048,488 |  |  |  |  |  |
| Average length of all-unigenes (nt) | 1487 |  |  |  |  |  |
| Length of N50 (nt) | 2034 |  |  |  |  |  |
